# Evaluating the efficacy of non-thermal microbial load reduction treatments of heat labile food components for in vitro fermentation experiments

**DOI:** 10.1371/journal.pone.0283287

**Published:** 2023-03-21

**Authors:** Andrew Paff, Darrell W. Cockburn

**Affiliations:** Department of Food Science, College of Agricultural Sciences, The Pennsylvania State University, University Park, Pennsylvania, United States of America; University of Illinois Urbana-Champaign, UNITED STATES

## Abstract

Increasingly, in vitro simulated colon fermentations are being used as a pre-clinical step to assess the impacts of foods and drugs on the gut microbiota in a cost-effective manner. One challenge in such systems is that they are potentially susceptible to the influences of contaminating microbes in test materials. Simulated gastric and intestinal digestion can relieve some of these concerns, however, live microbes may remain that can confound analysis. Autoclave treatment of test materials is the surest way to eliminate these microbes but presents problems when using heat labile components such as resistant starch. In this study, liquid chemical sterilant alternatives to moist heat sterilization were explored for treating pulse flours for use during in vitro simulated colon fermentation. Key attributes considered in chemical selection were accessibility, impact on treated food components, and effectiveness of the treatments for reducing microbial load. Three chemicals were selected for evaluation, bleach, alcohol, and hydrogen peroxide, at varying concentrations. Flours chosen for testing were from green lentil, field pea, chickpea, or sprouted green lentil. All treatments significantly reduced microbial loads, though there were still detectable levels of microbes after alcohol treatments. Furthermore, in vitro simulated colon fermentations of the treated pulses showed minimal difference from the untreated control both in terms of microbial composition and short chain fatty acid production. Scanning electron microscopy showed minimal impact of sterilization treatments on the gross structure of the pulse flours. Together these results suggest that bleach and hydrogen peroxide treatments can be effective nonthermal treatments to eliminate contaminating microbes in pulse flours without causing significant damage to starch and other fermentable substrates. This is thus also a promising treatment method for other starchy food substrates, though further testing is required.

## 1. Introduction

In vitro approaches are widely applied to microbiome research and are well suited to answer questions that necessitate an experimental setup that allows the researcher to exert a great deal of control over variables and compare a large number of treatments, both areas where an in vivo approach may fall short or be too costly [1–3]. Recent advancements in the development of in vitro approaches through application of 96 deep well plates, have boosted the appeal of these models, allowing their use as a high throughput method of colonic fermentation that maintains the structure of the microbiota [4, 5]. One area of control available to researchers using in vitro methods is the sterility of the input materials into the model. In some cases, such as studying fermented foods, it may be desirable to include fermenting organisms as potential probiotics. However, in most cases it is desirable to reduce or eliminate the native microbial community of substrates to be used in the colonic simulation, for instance, to minimize the influence that potentially different native microbial populations of the same food product produced in different locations may have on experimental outcomes. Preparation of materials for experimentation generally involves pressurized steam sterilization (autoclaving)–the most widely used form of sterilization [6]. While useful, steam sterilization has limitations in its applicability to heat labile items that require reduction of microbial loads. Furthermore, in starchy foods, sufficient heat with moisture results in heat moisture alterations, potential gelatinization of the starch, and variability in structural and physicochemical properties [7]. While this may simulate the cooking process, in some situations it is desirable to keep the starch intact, such as when investigating resistant starch. This starch is resistant to digestion by human enzymes, but is able to be fermented by bacteria in the colon where it can have beneficial effects on health [8]. However, this resistance requires the starch to remain in its semi-crystalline form, which is lost when it is heated sufficiently in the presence of water. During cooling, over time, the starch can re-crystallize in the process of retrogradation, and this retrograded starch is also resistant to digestion [9]. However, this structurally distinct form of resistant starch, known as Type 3, has different effects on the gut microbiota than the original granular resistant starch which is known as Type 2 [8]. Other notable heat induced alterations can also occur, such as Maillard browning or caramelization forming soluble and insoluble polymers [10]. Thus, there is a need for validated methods to reduce or eliminate microbes while not impacting downstream fermentation profiles of heat labile substrates.

A number of studies have evaluated alternatives to thermal microbial load reduction methods, such as non-thermal (“cold”) plasma, pulsed light, pulsed electric field, ozone treatment, and ultraviolet irradiation, among others, though these are typically evaluated from a food safety and sensory properties perspective as opposed to microbiome research with heat labile food components [11–13]. Additionally, these methods may require specialized equipment that may make them inaccessible to some researchers and in some cases may themselves have impacts on the structure of food components. Other non-thermal treatments include liquid and gas-based disinfectants/sterilants, though liquid-based methods are likely to be the most accessible. Many are routinely used for surface disinfection, such as classroom desks, and various options are available including alcohol, chlorine, glutaraldehyde, hydrogen peroxide, peracetic acid, and quaternary ammonium compounds [14]. However, studies related to their effectiveness have focused on their disinfection of hard surfaces [6, 15–17], rather than softer and more porous food components that may themselves be reactive with the treatment. One challenge with using liquid treatments is that some method of removing residual chemical before in vitro simulated colonic fermentation is necessary. This is easily achieved for insoluble substrates such as resistant starches using filtration, centrifugation, or dialysis, but may be more difficult for soluble substrates. A previous study performing a colonic simulation with resistant starch sources used 70% ethanol treatment to eliminate the innate microbes [3]. However, while this was sufficient to eliminate microbial load in isolated starches, effectiveness was not evaluated in more complex food matrices and thus, further testing is needed.

There is currently a gap in scientific understanding around the effectiveness of liquid sterilants as a non-thermal alternative to autoclaving of food materials prior to simulated colonic fermentation. This is particularly important for studying foods that are consumed in a raw form, including many supplements, to allow the study of the impact of these foods on the gut microbiota without interference from native microbes and without thermal destruction of heat labile components. Here we evaluate the efficacy of three different liquid chemicals (alcohol, hydrogen peroxide, and bleach) commonly accessible to labs at low prices in addressing the microbial load in green lentil, sprouted green lentil, chickpea, and field pea pulse flours. The selection of each were partly based on differences in intrinsic properties–alcohol is not sporicidal, bleach is alkaline (pH ~12), hydrogen peroxide is acidic (pH ~3.3). Other chemicals were considered but rejected, peracetic acid owing to acetic acid formation as a degradation product and ensuing potential interference with measuring acetic acid production during fermentation [18] and glutaraldehyde due to its reactivity with proteins [19]. We measure both the microbial load reduction achieved by these sterilants, their effect on starch structure as well as their downstream effects on simulated in vitro colonic fermentation of these flours. We find that both bleach and hydrogen peroxide are effective treatments and recommend their use by labs that are similarly exploring in vitro fermentations of heat labile food components, such as resistant starch.

## 2. Materials and methods

### 2.1 Materials

Liquid chemicals were 8.25% concentrated regular germicidal bleach (diluted with DI water to 1.0 and 1.5% sodium hypochlorite) (Clorox Company Commercial Solutions; Oakland, CA), 30% hydrogen peroxide (diluted with DI water to 2.0%) (EMD Millipore; Burlington, MA), and 90% reagent alcohol (diluted with DI water to 70%) (VWR International; Radnor, PA). All water used was type 1 DI water (Synergy UV-R, MilliporeSigma; Burlington, MA). Whole raw pulses comprised organic green lentil (*Lens culinaris*) (Arrowhead Mills; Boulder, CO), chickpea (*Cicer arietinum*) (Camellia Brand; Harahan, LA), and field pea (*Pisum sativum*) (Camellia Brand; Harahan, LA). Sprouted green lentil was produced in the lab, based on concepts covered in [20]. Sprouting entailed water washing the lentils, removing visible debris (i.e., rocks, sand) and soaking in water overnight. Soaked lentils were drained and rinsed. Rinsed lentils were spread out on damp paper towels and set into a dark place to prevent the sprouts from turning green and forming leaves. Damp paper towels were regularly changed and sprouting time ended when sprouts were around a quarter inch long (~3 days for lentils). To quickly dry, sprouted green lentil was thinly spread on a sheet and placed into a forced draft oven (Mechanical Oven, Lindberg/Blue M; Asheville, NC) at 50°C for 12 h, until dry. All pulses were made into pulse flours using a flour mill (WM2000, WonderMill Grain Grinder; Korea) on “coarse” setting. Resultant flours were passed through a #100 mesh sieve to minimize particle size exceeding 150 microns. The flour fraction passing through the sieve was kept and used as the pulse flour. All other chemicals used were from Sigma-Aldrich (St. Louis, MO) unless otherwise stated.

Human feces used for this research was sourced from a cryogenically stored lab stock of feces provided by a middle-aged male donor in 2020 located in the state of Pennsylvania (under approval of Penn State IRB; STUDY00013284). The lab stock of feces was produced by blending cryogenic buffer with the feces to produce a fecal slurry. Briefly, the fecal specimen had been returned within 1 h of defecation for processing and was immediately transferred into an anaerobic chamber, diluted with 3 volumes of cryogenic buffer (136.89 mM NaCl, 2.68 mM KCl, 10.14 mM Na_2_HPO_4_, 1.76 mM KH_2_PO_4_, 11.39 mM L-Cysteine-HCL monohydrate, 0.2% Resazurin, 20% glycerol) [21], thoroughly blended on high setting (38BL54, Waring; Torrington, CT), and aliquoted into pre-labeled 2 mL cryotubes (VWR, Wayne, PA). Cryotubes were removed from the anaerobic chamber and stored at –80°C until further use.

### 2.2 Pulse sterilization treatments

The liquid treatment process consisted of measuring appropriate pulse flour into falcon tubes and suspending in liquid chemical to 4% weight/volume. Flour and liquid were thoroughly mixed by vortexing. The mixed tubes were then placed horizontally on an orbital shaker (VWR; 97109–890) set at 218 RPM and incubated at room temperature (~21°C) for 24 h. Dry autoclaved flours and untreated flours were both suspended and thoroughly mixed in type 1 DI water at 4% weight/volume and added into the centrifuge. All flours were centrifuged for five minutes at 4,000xg (Allegra X-14R, Beckman Coulter; Brea, CA). The supernatant was poured off and type 1 DI water was added and thoroughly mixed to produce a 4% weight/volume slurry. The washing process with type 1 water was repeated ten times, prior to use in subsequent experiments.

Based on preliminary experimentation, bleach at 1.0% and 1.5% (active chlorine 0.95 and 1.43% respectively), hydrogen peroxide at 2.0%, and reagent grade alcohol (81.0–82.0% ethanol, 3.8–4.5% methanol denaturant, 4.0–5.0% 2-propanol, water balance) at 70% volume/volume were selected for further analysis. Additionally, dry autoclaved (121°C, 25 minutes), and untreated versions of all flours were included as controls in all experiments.

### 2.3 Viable plate counts

All viable counts and fermentations were performed using the RUM media, which is formulated to permit growth of many anaerobes of the human colon, including the fastidious RS degrader *Ruminococcus bromii* and was prepared as previously described [3, 22]. Media was aliquoted and moved into anaerobic chamber (Coy Laboratory, Grass Lake, MI) outfitted with moisture, and hydrogen sulfide removal column, and anaerobic gas monitor for oxygen and hydrogen level. Chamber gases are 5% hydrogen, 8% carbon dioxide and the balance nitrogen.

Filter sterilized 2X concentrated RUM and autoclaved 2% agar were combined 50/50 volume/volume and poured into petri dishes. Cooled petri dishes were moved into the anaerobic chamber for 24 h prior to plating samples. Untreated, untreated and washed, and treated and washed flours were resuspended in anaerobic Phosphate Buffer Solution (PBS), at 10% weight/volume by vortexing. All treated samples were plated immediately. Untreated samples were either plated immediately (As is) or subjected to the same washing procedure as the treatments and then plated (Washed). To simulate potential microbial population expansion during processing, another set of untreated samples was resuspended at 4% weight/volume as described for the treatments, incubated for 24 h, washed, resuspended at 10% weight/volume and then plated (Incubated, Washed). Appropriate dilutions for each sample (up to 10^7^-fold) were plated in triplicate (0.1 mL) and incubated at 37°C in an anaerobic incubator for 24 h.

At 24 h, plates were removed from the anaerobic chamber and counted. Averages of each triplicate were used in determining the colony forming units (CFU) at a given plating dilution. Countable average CFU range used was 25 to 200 CFU [23] for a limit of detection of 2.5x10^3^ CFU/g pulse flour.

### 2.4 Fermentation experiments

Following sterilization and washing, pulse flours were transported into the anaerobic chamber and then resuspended in anaerobic RUM media to make a 2% weight/volume pulse flour slurry. Fermentation media with fecal microbes was generated in a similar manner, except 0.125 mL fecal sample slurry was incorporated for a resulting 1% fecal volume/volume solution. Fermentations were carried out with each of the pulse flours undergoing each of the sterilization treatments, along with an unsterilized control, with and without the addition of fecal microbes.

Fermentations were performed either statically in deep 96 well plates (Axygen; P-DW-20-C-Covance) or in deep 96 well plates with shaking (500 RPM; BioShake iQ, Bulldog Bio, Portsmouth, NH) to represent variations of simulated colon fermentation procedures [5]. All fermentations were performed in biological triplicate. Fermentation vessels each received 1.5 mL of the appropriate RUM + pulse slurry in triplicate. After 24 h, all fermentation vessels were removed from the anaerobic chamber and subjected to centrifugation (15 minutes at 2,500xg). The supernatant was removed and both pellet and supernatant were frozen at –80°C until further use downstream.

### 2.5 Sequencing of the 16S V4 region of microbial communities

One replicate of each fermentation was analyzed for microbial community composition. Fermentation pellets were processed using QIAmp PowerFecal DNA kit following the manufacturer’s instructions except that samples were homogenized in a mini-BeadBeater 96 unit (Biospec, Bartlesville, OK; Mfr. No. 1001) for 5 minutes at 3800 RPM. Extracted DNA was stored at –20°C until further use.

Amplification of the 16S V4 region was performed using the Accuprime HiFi polymerase (Invitrogen) in a 1-step PCR protocol using primers (513F, 806R) that incorporate Illumina adaptors and barcodes [24]. A thermocycler (Eppendorf 5331) running the following program sequence: 2 minutes at 95°C, then 30 cycles of 20 seconds at 95°C, 15 seconds at 55°C, and 5 minutes at 72°C was used for amplification. Amplified PCR reactions were checked for purity on an agarose gel and rerun, as necessary. Samples were then normalized with Invitrogen SequalPrep Normalization Plate [96] kit (A10510-01). The normalization protocol supplied with the kit was followed for performing standard elution. Normalized liquid samples were transferred into storage containers and sent to Pennsylvania State University Genomics Core Facility for MiSeq Illumina 250x 250 paired-end sequencing.

### 2.6 Analysis of microbial community composition

Demultiplexed sequences of the V4 region of 16S rRNA were subjected to quality control and clustering into de novo generated Operational Taxonomic Units (OTUs) using Mothur version 4.1.1 and consulting the SOP of Schloss et al. accessed October 2021 [24]. RDP version 18, released July 2020, was used to identify OTUs down to genus level. Outputs from Mothur were a Taxonomy file, Fasta file, and OTU table.

BLAST (Basic Local Alignment Search Tool) from NCBI (National Center for Biotechnology Information) was used to compare the representative sequence from each OTU generated by Mothur to the 16S Microbial database to identify taxonomy to species level, if at least 97% of sequence identity was shared, otherwise genus level identification was maintained. Taxonomy to species level was added to the Mothur taxonomy file. A phylogenetic tree of the OTU sequences was generated using the program FastTree and the fasta file generated by Mothur [25].

Analysis of microbial sequences was performed in R using the phyloseq package and other compatible packages as indicated [26]. Multiple indices of both alpha (Shannon and Inverse Simpson) and beta diversity (Aitchison, Bray-Curtis, and Weighted UniFrac) were run to compare community diversity within samples and between samples, respectively. The DivNet package was used to obtain Shannon and Inverse Simpson values at the genus level [27]. The alpha diversity values were analyzed by ANOVA, investigating the pulse flour, sterilization method and agitation as factors. Non-significant factors were removed from the final model and pairwise comparisons of the sterilization treatments with the unsterilized control were examined via the Dunnett test. Benjamini-Hochberg procedure was used to address false discovery rate with multiple pair-wise comparisons [28]. Beta diversity analysis was performed at the genus taxonomic level. Aitchison distance was determined by a centered-log transformation of data with the Microbiome package [http://github.com/microbiome/microbiome], followed by determination of the Euclidian distance. The Bray-Curtis distance was computed without prior transformation of the data using the vegdist function of the Vegan package [https://cran.r-project.org/web/packages/vegan/index.html]. The weighted UniFrac distance was calculated using the phyloseq package following rarefaction of the dataset. All beta diversity indices were plotted, via principal coordinate analysis to evaluate for clustering based on treatment. Each of the three beta indices used the adonis2 function from the Vegan package to perform a PERMANOVA test for overall significance of treatments, while the pairwiseAdonis2 package [https://github.com/pmartinezarbizu/pairwiseAdonis] was used to test for individual treatment difference significance compared to the unsterilized control [29]. The P. adjust function, applying the Benjamini-Hochberg procedure, was used to address false discovery rate of pairwise comparisons.

Differential abundance was evaluated using three statistically distinct methods, LEfSe, DESeq2 and ANCOM-II [30–32]. The OTUs were first filtered for a minimum abundance (0.001% of reads) and prevalence (5% of samples). DESeq2 and ANCOM-II were performed with their respective packages in R, while LEfSe was conducted by first converting reads to relative abundances and submitting to the Huttenhower lab’s Galaxy server (https://huttenhower.sph.harvard.edu/galaxy/). Each method was run with default settings and used to examine the differences between each of the treatments and the unsterilized control, using the Benjamini-Hochberg method to correct for multiple comparisons.

### 2.7 Measurement of short chain fatty acids

Butyrate and acetate concentrations were measured by UV absorbance using a Thermo Scientific Dionex ICS-5000+, running Chromeleon software version 7.2.6 and set-up for measuring organic acids with a UV detector operated at 210 nm wavelength. An ion exclusion column (Aminex; HPX-87H; 300x7.8 mm) at 50°C was used for separation with an isocratic mobile phase of 5 mM sulfuric acid at a flow rate of 0.45 mL per minute. Calibration curves were produced by making desired organic acid concentrations (100, 50, 25, 12.5, 6.25, 3.13 mM). All standards had 5 mM sulfuric acid present. Contribution of acids by RUM media was measured and subtracted from samples. Supernatants from pulse fermentation were mixed 1:1 on a volume basis with 10 mM sulfuric acid. Sample run time was 60 minutes, with butyrate and acetate retention times at approximately 28 and 20 min, respectively.

Determined organic acids concentration from fermentation samples were averaged between triplicate runs and then analyzed in R by ANOVA, using the pulse flour, sterilization method and agitation as factors. Non-significant factors were removed from the final model and pairwise comparisons of the sterilization treatments with the unsterilized control were examined via the Dunnett test. False discovery rate with using pair-wise comparison were addressed with the Benjamini-Hochberg procedure.

### 2.8 Scanning electron microscopy: Sample production and handling

Washed pulse samples were suspended in PBS following sterilization treatment and were dried in a forced draft oven (Blue M, New Columbia, PA) overnight at 42°C and allowed to cool in the oven. Dried samples were powdered and stored until further use. Double sided carbon tabs (Ted Pella, Redding, CA; 16084–1) were placed on standard pin stub mounts [Ted Pella; 16111]. Carbon tape on the stub was pressed into powder to coat the stub entirely. Loose powder was removed by spraying the sample with canned air. To run a sample on the SEM, stub with sample was placed into a charge reduction sample holder (Phenom World; Waltham, MA). SEM was then performed on a Phenom G2 Pro instrument (Phenom World; Netherlands). Images at various locations and magnifications were taken at 5 kilovolts.

## 3. Results and discussion

### 3.1 Effect of sterilization treatments on native pulse microbial load

**Fig 1:**
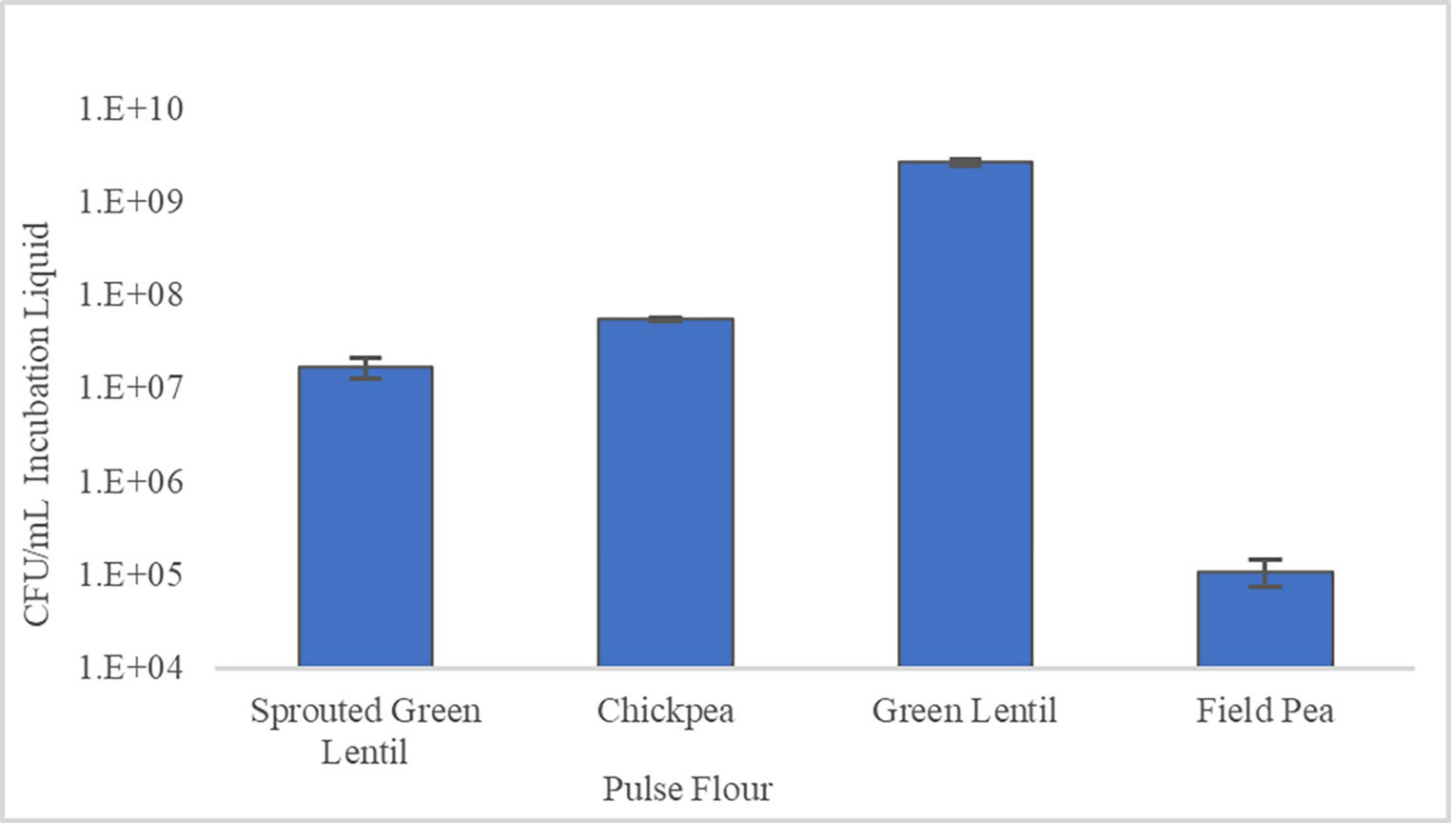
Viable microbial counts of overnight water incubated pulse flours. For each pulse, 0.5 g of each pulse flour was aerobically incubated overnight with agitation in 12.5 mL type 1 DI water. After washing with type 1 DI water, pulses were plated on anaerobic RUM media and incubated anaerobically for 24 h at 37°C.

Viable plate counts on RUM media were used to evaluate the effectiveness of sterilization treatments on native pulse microbial load. Plates were incubated anaerobically at 37°C for 24 h to match conditions used for simulated colonic fermentation. In Table 1 the microbial loads present in the pulse flours (“As is” control samples) range from below the limit of detection in field pea to 2.5 x10^5^ CFU/g in the green lentil flour. Administering a simple 10x water rinse to “as is” control samples achieves an approximate 1-log reduction. However, as can be seen in Fig 1, hydrating the flours and incubating them, simulating processing to prepare for colonic fermentation experiments, results in a substantial expansion of the microbial populations by up to 5-logs.

**Table 1:** Viable microbial counts of controls and treated pulse flours plated on RUM media and incubated anaerobically for 24 h at 37°C.

Treatment	Sprouted Green Lentil (CFU/g)	Chickpea (CFU/g)	Green Lentil (CFU/g)	Field Pea (CFU/g)
Autoclave	<2.5x10^3^	<2.5x10^3^	<2.5x10^3^	<2.5x10^3^
1.0% Bleach	<2.5x10^3^	<2.5x10^3^	<2.5x10^3^	<2.5x10^3^
1.5% Bleach	<2.5x10^3^	<2.5x10^3^	<2.5x10^3^	<2.5x10^3^
70% Alcohol	<2.5x10^3^	<2.5x10^3^	4.9x10^4^	<2.5x10^3^
2.0% H2O2	<2.5x10^3^	<2.5x10^3^	<2.5x10^3^	<2.5x10^3^
“As is”	1.8x10^5^	6.6x10^4^	2.5x10^5^	<2.5x10^3^
Washed	6.3x10^4^	<2.5x10^3^	4.3x10^4^	<2.5x10^3^

Autoclaving, 1.0% bleach, 1.5% bleach, and 2.0% hydrogen peroxide were all equally effective at ensuring a microbial load reduction to the limit of detection of the assay, <2.5x10^3^ CFU/g pulse flour [Table 1]. Seventy percent alcohol was the only liquid chemical not able to ensure a microbial load reduction to <2.5x10^3^ CFU/g in all pulse flours, with green lentil having a load of 4.9x10^4^ CFU/g flour after alcohol treatment and washing. Although 70% alcohol was not as effective as the other treatments, it is still worth acknowledging at least a 4-log reduction in microbial load, compared to incubating in water alone (Fig 1). In many cases this may be sufficient as a typical colonic fermentation introduces bacteria at a concentration of between 10^6^ to 10^7^ CFU/mL, while a 2% flour at 4.9x10^4^/g would only introduce around 10^3^ bacteria per mL, representing between 0.01 and 0.1% of the bacteria in the fermentation [33]. This may still be problematic if rare taxa are under consideration, however.

### 3.2 Pulse bacterial communities during endogenous fermentation

#### 3.2.1 Endogenous pulse community composition

**Fig 2:**
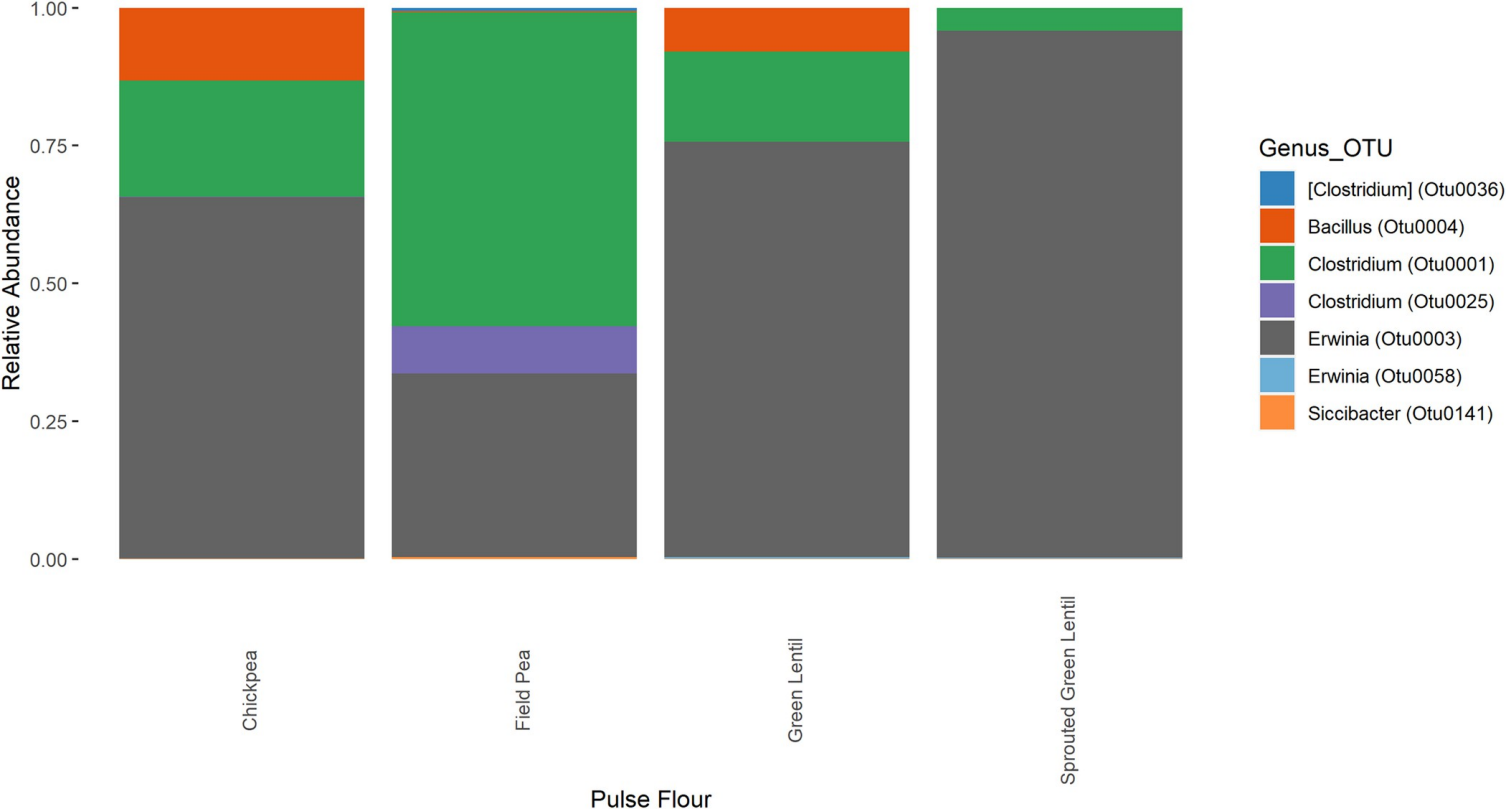
Relative abundance of endogenous microbes in pulse flours. Pulses were resuspended in RUM media and incubated anaerobically at 37°C without addition of fecal microbes. Each color corresponds to an OTU present at >0.1% relative abundance. Each bar represents a different untreated pulse flour.

To explore what microbes present in the pulses could impact colonic fermentations, endogenous fermentation without any feces added were conducted in the same media used to simulate colon conditions. Identification of OTUs is at the genus taxonomic level, owing to difficulty in identifying microbes down to the species level using the V4 region of the 16S rRNA gene alone [34, 35]. Seven OTUs were found to be present at greater than 0.1% of the relative abundance in each of the pulse flours (Fig 2). Of the seven OTUs, OTU0003 (*Erwinia*) had the highest relative abundance in chickpea (65.5%), green lentil (75.3%), and sprouted green lentil flour (95.5%), while OTU0001 (*Clostridium*) had the highest relative abundance in field pea flour (57.1%). OTU0004 (*Bacillus*) and OTU0025 [*Clostridium*] also contributed notably to chickpea flour [13.2%] and green lentil flour (7.9%), or field pea flour (8.5%), respectively. Lastly, OTU0141 (*Siccibacter*), OTU0036 (*Clostridium*) and OTU0058 (*Erwinia*) were the final OTUs detected above the 0.1% threshold, with relative abundance in all pulses below 1%. Certain species of *Erwinia*, such as *Erwinia tasmaniensis*, are known to be pectolytic bacteria, able to be isolated from plants and associated with vegetable spoilage [36, 37]. Therefore, it is not surprising to find the *Erwinia* genera present in the endogenous pulse community. As for the remaining identified taxa, *Bacillus* (OTU0004), *Siccibacter* (OTU0141), and *Clostridium* (OTU0001, OTU0025, and OTU0036), various species within each genus are associated with plants. The *Bacillus* genus includes facultative psychrotrophic species, such as *B*. *wiedmannii*, originally called FSL W8-0169 and considered a food spoilage organism widely distributed in natural environments [34, 35]. The *Clostridium* genus includes *C*. *puniceum*, a microbe able to be isolated from plants and associated with vegetable spoilage [38, 39], *C*. *aurantibutyricum*, an anaerobe capable of acetate and butyrate production and able to utilize starch [40, 41], and *C*. *celerecrescens*, an anaerobic cellulolytic bacterium, originally isolated from cellulose-enrichment culture inoculated with cow manure [42]. Finally, the genus *Siccibacter* includes facultative anaerobes able to be isolated from plant material, such as *S*. *colletis* [43]. Given the association with plants of members within each of the five remaining taxa, it is not surprising that members of these taxa were also contributors to the endogenous pulse community.

#### 3.2.2 Organic acid production

We investigated the ability of these microbes to produce organic acids from the pulse flour under simulated colonic conditions as this is often a key measurable outcome from these experiments. Field pea fermentation produced a small amount of butyrate, while all four pulses showed an increase in acetate level that was statistically significant (Table 2). As *Erwinia* is capable of acetate production and was present in all samples and was likely the main contributor [44]. The main contaminant in field pea was *Clostridium*, differing from that of the other pulse flours (Fig 2). Many Clostridium species can produce butyrate, and they are the likely source of butyrate in the field pea fermentations, however, the butyrate concentrations (Table 2) were not significantly different from the media only control. This suggests endogenous pulse microbes by themselves would not directly interfere with measuring butyrate production during simulated colonic fermentation, though their interactions with other microbes could still potentially indirectly impact butyrate production [40]. Other potential organic acids such as succinate, formate or lactate were not present above the limit of detection.

**Table 2:** Butyrate and acetate concentrations produced by endogenous pulse flour microbes in untreated pulse flours following a 24-h anaerobic endogenous fermentation at 37°C.

Pulse Flour	Butyrate (mM)	Acetate (mM)
Chickpea	Not Detected	29.8 ± 2.5 ***
Field pea	4.0 ± 8.9	23.7 ± 3.9 ***
Green Lentil	Not Detected	27.7 ± 0.9 ***
Sprouted Green Lentil	Not Detected	31.9 ± 6.4 ***

* *p* < 0.05

** *p* < 0.01

*** *p* < 0.001

### 3.3 Simulated in vitro colonic fermentation

#### 3.3.1 Community composition

**Fig 3:**
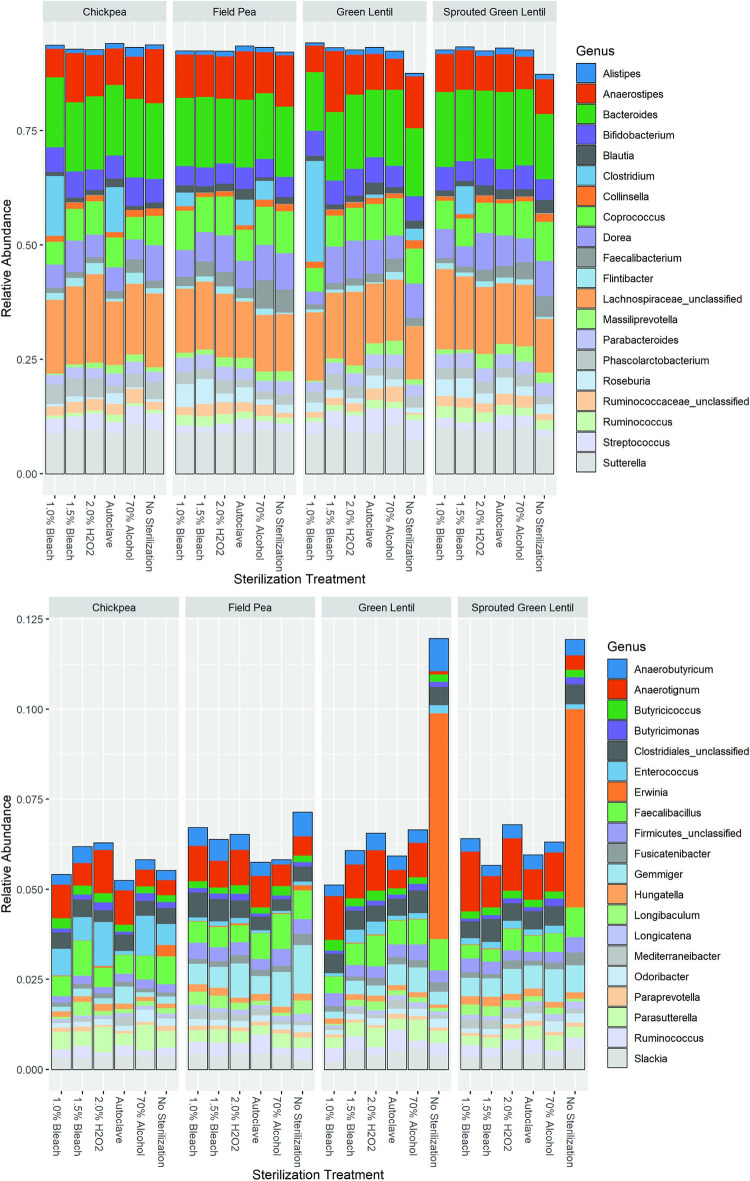
Relative abundance of microbes in simulated in vitro colonic fermentations run for 24 h at 37°C with fecal addition. Each set of treatments is grouped by pulse identity. (A) Relative abundance of the top 20 genera. (B) Relative abundance of the next 20 most abundant genera.

To analyze the impact of sterilization on microbial communities during simulated colonic fermentation, such fermentations were performed with both unsterilized pulses and pulses that had undergone the various sterilization treatments. The relative abundance of the top 20 genera (average across all pulses and treatments) (Fig 3A) did not differ greatly from one treatment to another or from one pulse to another with the exception that the *Clostridium* genus seemed to expand in a random subset of treated samples, averaging 0.69% of the community across the unsterilized pulse fermentations, reaching a high of 22% of the community in the 1% bleach treated green lentils. It is not clear what would be driving this expansion in some treated samples as the expansion seems to be mainly due to OTU0017, which was not found in the pulses. Examining the relative abundance of the next 20 most abundant genera (Fig 3B) showed a clear expansion of *Erwinia* in the unsterilized samples of both green lentil (6.3% of the community) and sprouted green lentil flour (5.5% of the community). This *Erwinia* was also present, though at lower levels, in the unsterilized chickpea (0.32% of the community) and field pea (0.14% of the community) fermentations. This is compared to an average of 0.02% of the community across all treated samples. Given its association with the pulse community it seems highly likely that this *Erwini*a is derived from these pulse samples and can thrive, even when competing with the high abundance of microbes in the fecal samples. However, the sterilization methods tested here, appear to greatly reduce the relative abundance of this bacterium.

#### 3.3.2 Differential abundance analysis

**Fig 4:**
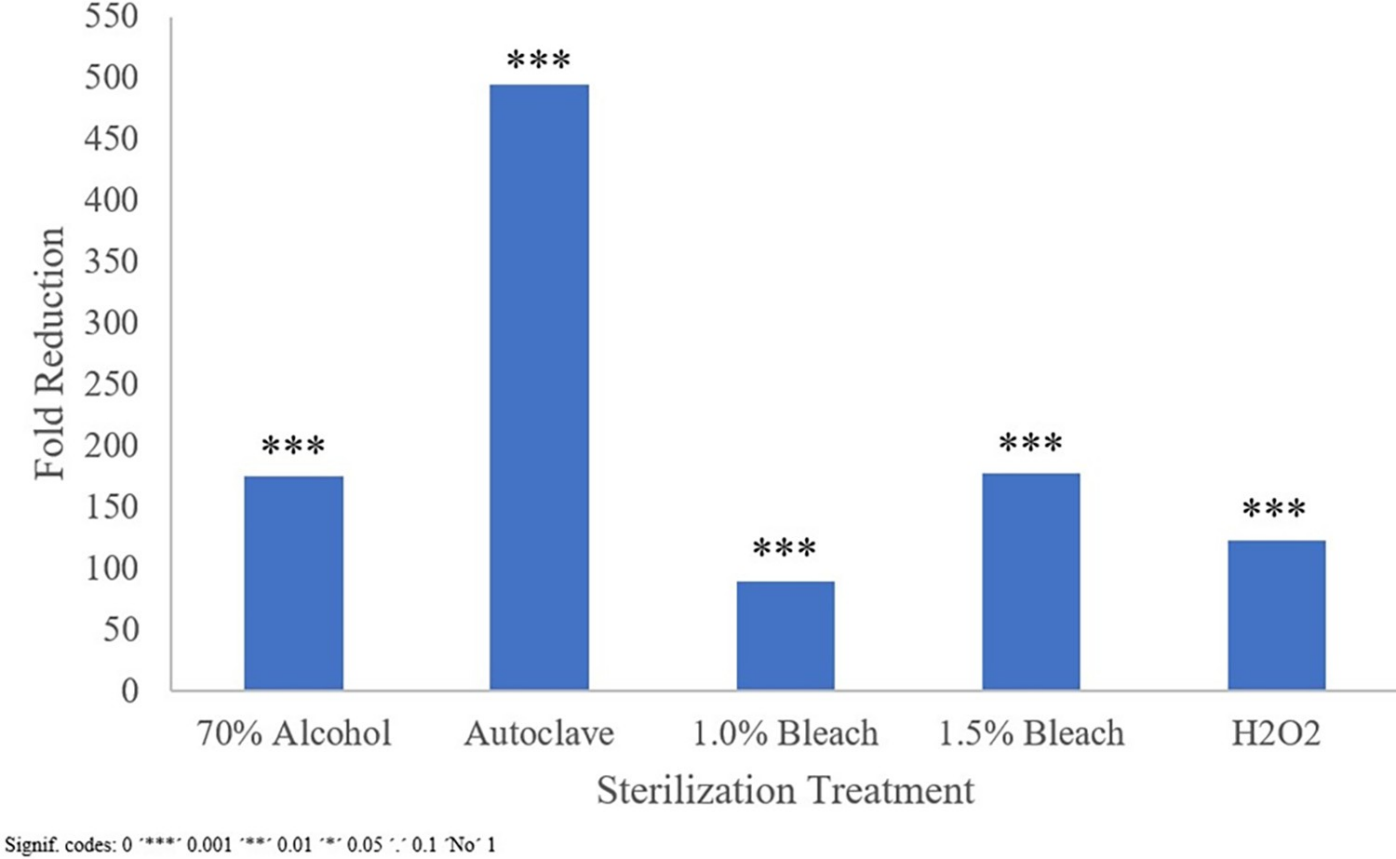
DESeq2 differential abundance of OTU003 (*Erwinia*) in treated pulse flours following simulated in vitro colonic fermentation for 24 h at 37°C with feces added. All pulse flour types are grouped together by their treatment and comparisons are made against the unsterilized control The *p*-values from DESeq2 were corrected for multiple comparison with the false discovery rate method, * indicating corrected *p* < 0.05, ** corrected *p* <0.01, *** corrected *p* < 0.001.

**Fig 5:**
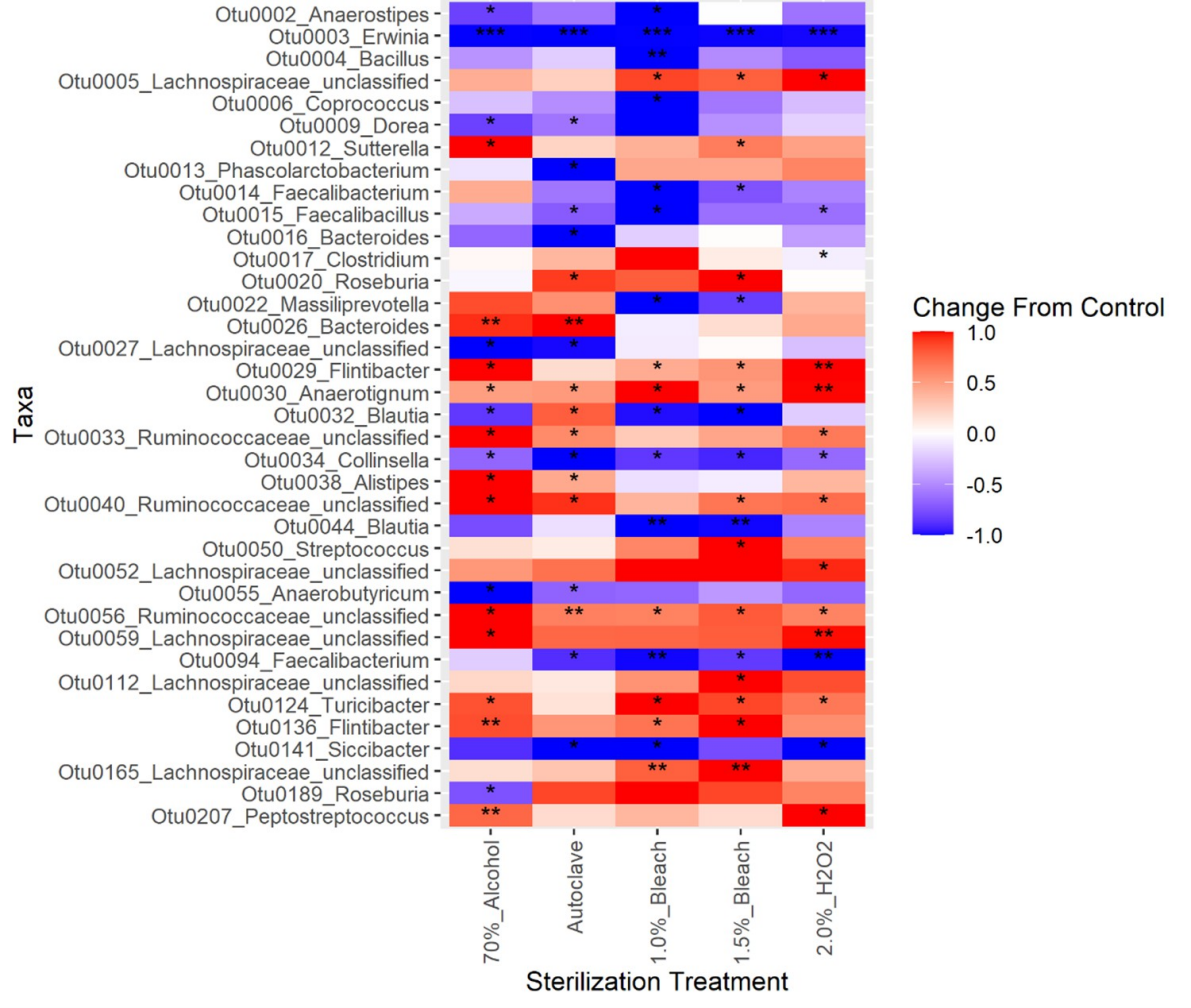
Heatmap of differential abundance for pulse flour treatments that have undergone 24 h simulated in vitro colonic fermentation at 37°C. An asterisk indicates significance (FDR corrected *p* < 0.05) for one differential abundance test—ANCOM-II, LEfSe, or DESeq2. Two asterisks indicate significant differences as determined by two of the tests, three asterisks indicate significant differences by all three methods. Each treatment group includes all four pulse types. Red indicates an increase, while blue indicates a decrease in differential abundance. OTU0003 (*Erwinia*) is the primary microbe that changed in differential abundance by decreasing compared to unsterilized pulse flours.

As *Erwinia* (OTU0003) had among the highest relative abundance in the pulse flours evaluated and was shown capable of impacting the community structure during fecal fermentations (Fig 3B), evaluation of differential abundance focused on *Erwinia* in unsterilized pulses compared to treated pulse samples. DESeq2 analysis, indicated that all treatments statistically significantly reduced relative abundance of *Erwinia* (α = 0.05). The magnitude of the reduction ranged from 90-fold in 1.0% bleach to 500-fold in the autoclave treatment (Fig 4), corroborating the substantial changes in relative abundance seen in Fig 3B. Two other differential abundance methods, ANCOM-II and LEfSe, both also found significant reductions in *Erwinia* abundance by all sterilization treatments (Fig 5). Of the other bacteria found in the pulse flours, only *Bacillus* (OTU0004) and *Siccibacter* (OTU0141) were also found in the fecal fermentations, though at much lower levels. *Bacillus* was significantly reduced with 1.0% Bleach according to two of the differential abundance methods, while *Siccibacter* was found to be significantly reduced in the autoclave, 1.0% bleach and 2% hydrogen peroxide treatments by only one of the differential abundance methods. Three OTUs were found to have been significantly changed by all the sterilization treatments by at least one of the differential abundance methods. These were *Anaerotignum* (OTU0030), *Collinsella* (OTU0034) and an unclassified Ruminococcaceae member (OTU0056). Each of these are commonly found members of the gut microbiota and were not found in the pulse only samples. Of these the *Anaerotignum* and the unclassified Ruminococcaceae member were found to increase in sterilized pulse fermentations relative to the unsterilized, suggesting they may have been suppressed by the presence of pulse contaminants. In contrast the *Collinsella* OTU was more abundant when using unsterilized pulses, suggesting it may have had a positive interaction with one or more of the pulse contaminating organisms. While, in most cases these changes were only found to be significant by one of the differential abundance methods, the consistency across the sterilizations suggest that this is a real effect. Overall, other than *Erwinia*, no OTU was found to be significantly different from the unsterilized samples by all three of the differential abundance methods, suggesting only relatively minor changes to non-pulse derived microorganisms of the sterilization treatments.

#### 3.3.3 Alpha and beta diversity

Both Shannon and Inverse Simpson indices were used to characterize the effects of sterilization treatment on alpha diversity in simulated colonic fermentations with the pulses. Aitchison, Bray-Curtis, and weighted UniFrac indices were used to calculate beta diversity.

**Fig 6:**
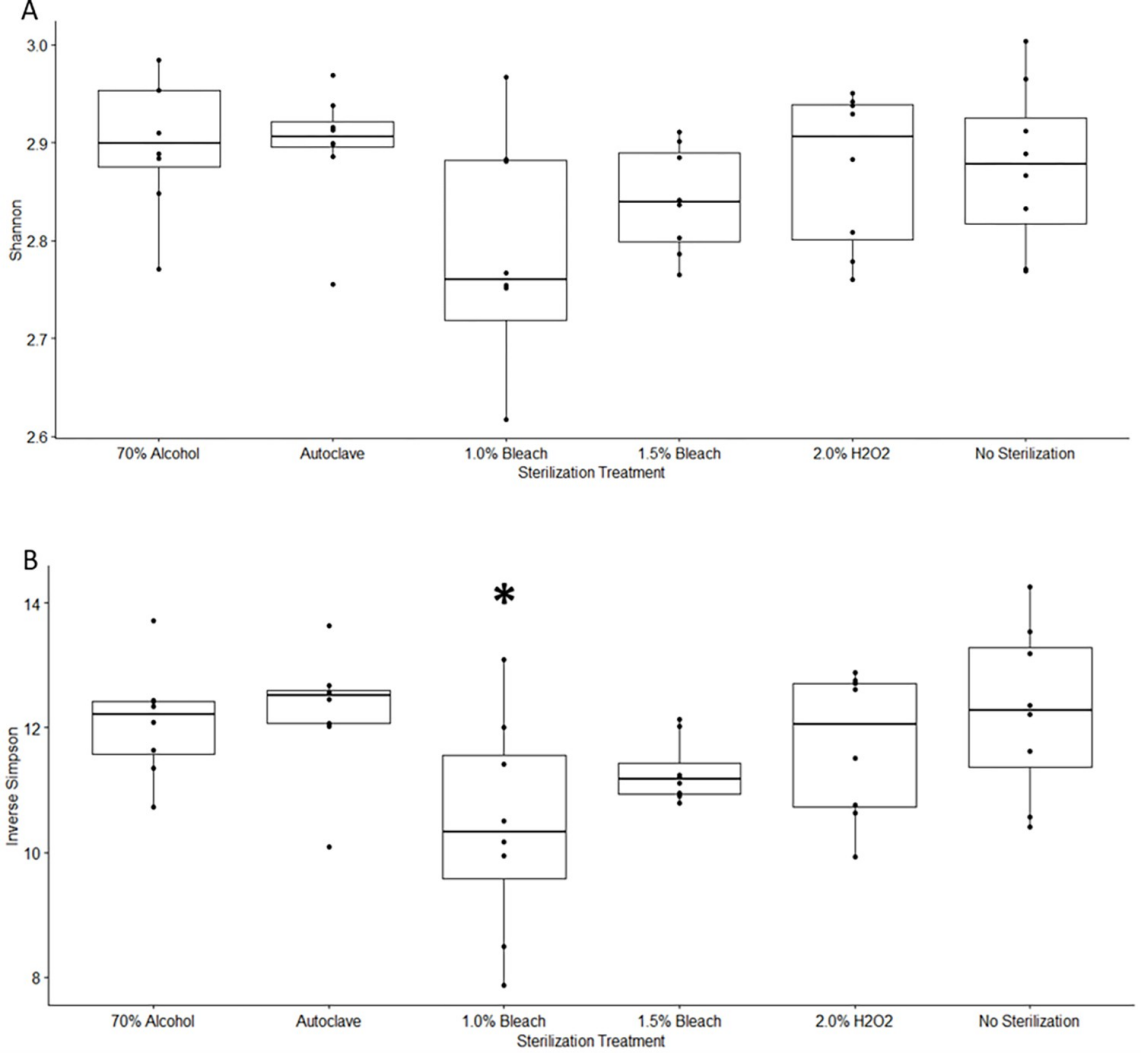
Shannon and Inverse Simpson diversity of simulated in vitro colonic fermentation of six different pulse flour treatments. Each treatment includes all four pulse types. (A) Shannon index, pairwise comparison of each treatment against untreated. (B) Inverse Simpson index, pairwise comparison of each treatment against untreated. * indicates *p* < 0.05.

All pairwise comparisons against the unsterilized pulse fermentations using Shannon index were not significant at α = 0.05 (see Fig 6A). In contrast, all pairwise comparisons using Inverse Simpson index were not significant, except for 1.0% bleach (*p*-value 0.0107) which indicated lower diversity (Fig 6B). The Simpson index bias to consider evenness suggests the 1.0% bleach community relative abundance is less evenly distributed among species, although it is possible that this is a type 1 error given the 1.5% bleach did not agree and the Shannon index did not indicate a significant difference for 1.0% bleach. However, the 1.0% bleach, also had the lowest (though non-significant) mean Shannon diversity and the greatest variability in diversity. It is possible that this indicates that the 1.0% bleach is not quite a strong enough sterilant to produce consistent results.

**Fig 7:**
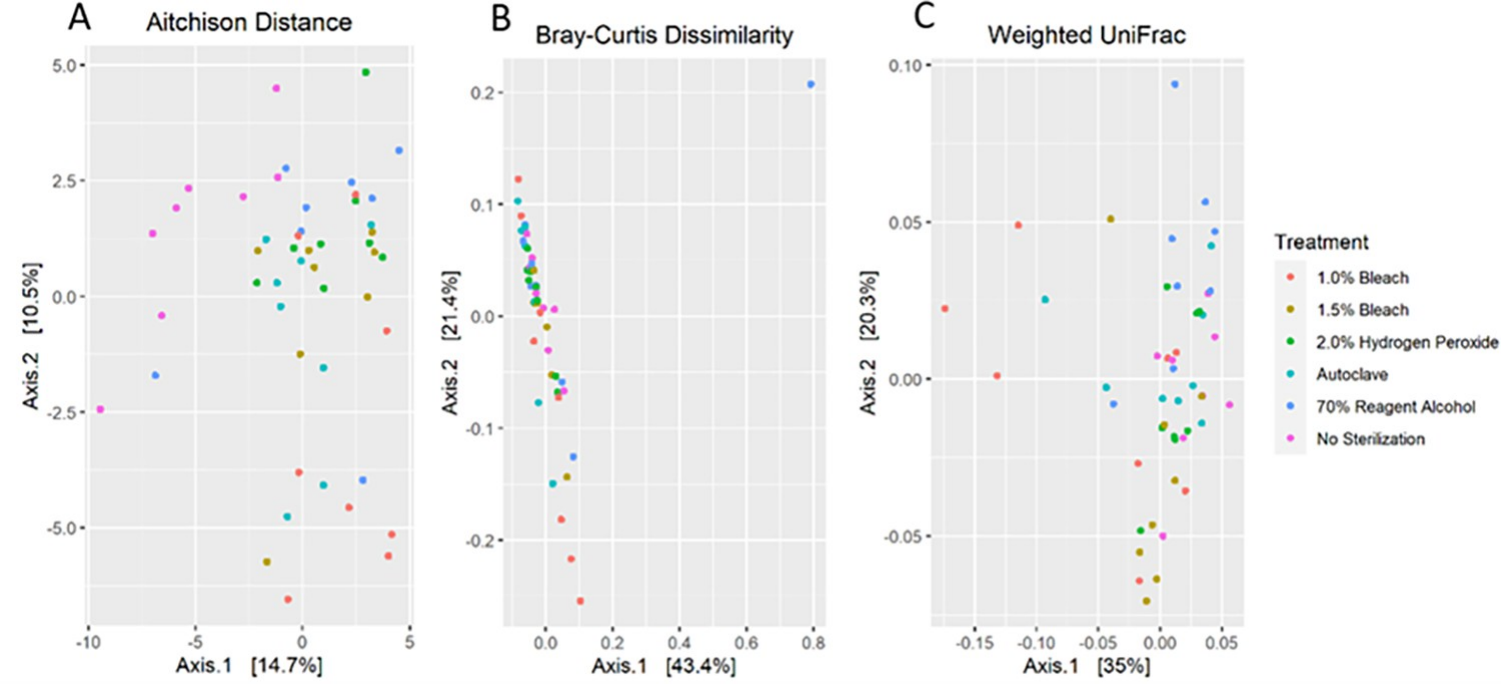
Beta diversity of 24 h simulated in vitro colonic fermentations performed at 37°C on treated pulses with feces added. (A) Aitchison; (B) Bray-Curtis; (C) Weighted UniFrac beta indices.

For beta diversity, a combination of Euclidean (distance) and non-Euclidean (dissimilarity) beta indices should be considered, as Euclidean can suffer from a paradox of two samples with no species in common being indicated more similar than two samples with many species in common, depending on the dataset [45, 46]. Distance quantifies differences between objects without considering what the objects have in common, vs dissimilarities scale objects in accordance with comparing the difference between objects to what they have in common [45]. Here, three beta indices were used to compare sterilized pulses against the unsterilized in the simulated in vitro colonic fermentations, two Euclidian based (Aitchison and Weighted UniFrac) and one non-Euclidean (Bray-Curtis). The results of a pairwise PERMANOVA test of these indices are shown in Table 1, while ordinations are shown in Fig 7.

Considering all pairwise comparisons between treating pulse flours with a sterilization method vs unsterilized (Table 3), all comparisons for Aitchison and weighted UniFrac were statistically significant. Bray-Curtis, although not significant, did not greatly exceed the 0.05 cut-off used.

**Table 3:** Adjusted P-values from PERMANOVA tests of beta diversity indices looking at simulated in vitro colonic fermentations. Each treatment groups all four pulse types together.

Treatment	Aitchison	Bray-Curtis	Weighted UniFrac
Autoclave vs no sterilization	0.00375 **	0.07500 .	0.01500 *
Alcohol vs no sterilization	0.00600 **	0.09938 .	0.01500 *
1.5% Bleach vs no sterilization	0.00375 **	0.07500 .	0.04500 *
1.0% Bleach vs no sterilization	0.00375 **	0.07500 .	0.01500 *
H2O2 vs no sterilization	0.00375 **	0.07500 .	0.04650 *

Significance codes

*** 0.001

** 0.01

* 0.05,. 0.1

Overall, the three beta diversity tests indicate the fecal fermentation communities of all sterilized pulses are at least trending to being different from the unsterilized ones, whereas many of the sterilization treatments do not significantly differ from one another and do not visually separate in a principal coordinate analysis plot (Fig 7). This suggests that it is primarily the microbes introduced from the pulses that are driving the differences seen here. However, given that the Bray-Curtis method did not detect any significant differences, the magnitude of the overall community differences appears to be relatively small.

#### 3.3.4 Organic acid production during simulated colonic fermentations

**Fig 8:**
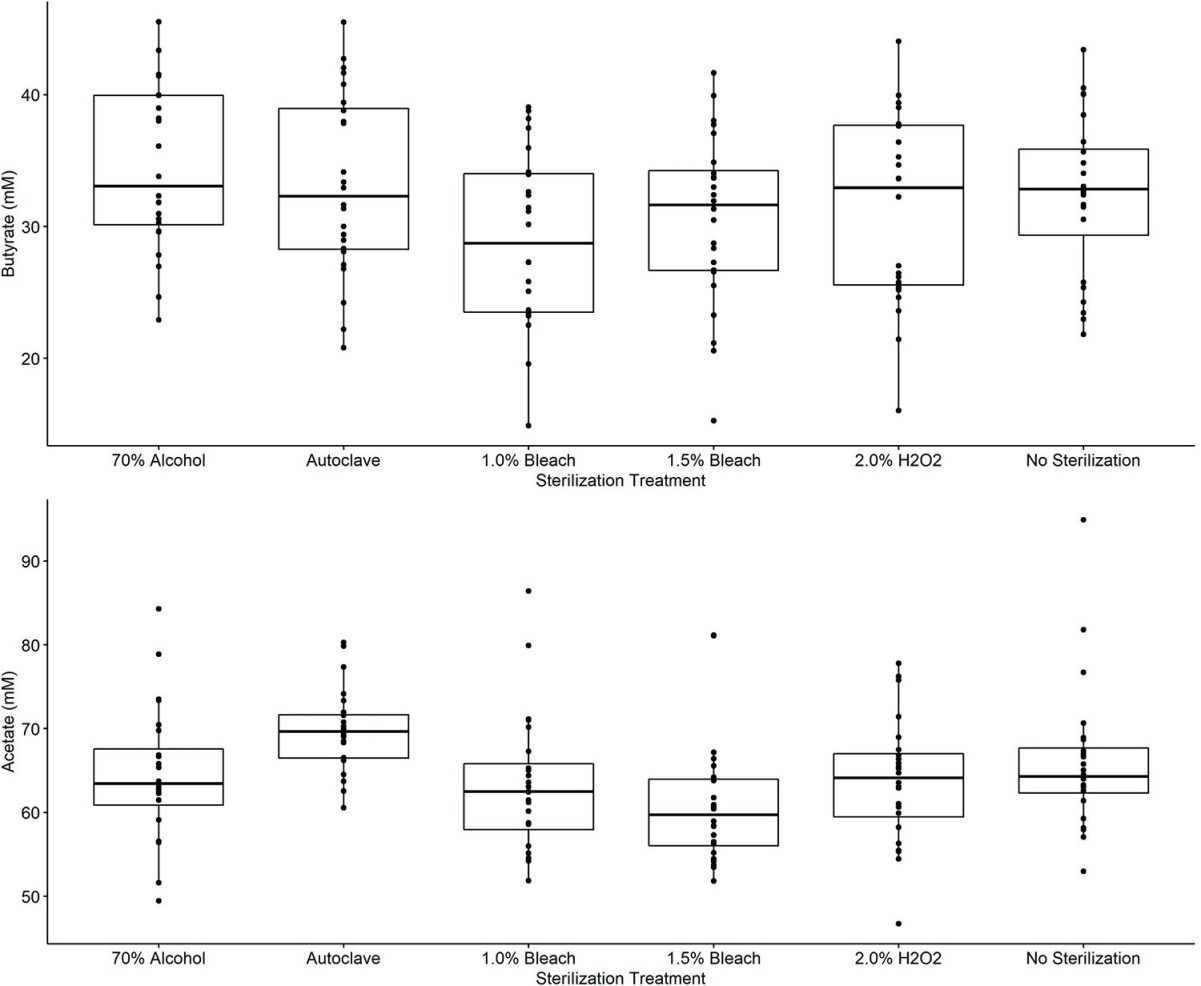
Comparison of (A) butyrate concentrations and (B) acetate concentrations following simulated in vitro colonic fermentations at 37°C for 24 h on treated pulse flours with fecal inclusion in RUM media. No significant differences were found in butyrate or acetate concentrations between sterilized and unsterilized fermentations.

Beyond just impacts on the community structure, it is important to evaluate community function. As a key measure of this, the concentration of organic acids produced, particularly acetate and butyrate was investigated, following simulated in vitro colonic fermentations. Note that due to a tight co-elution of a media component with propionate it was not possible to accurately assess its concentration in this model using HPLC. While acetate and butyrate production levels differed by the pulse substrate used, there was no significant effect of the sterilization treatment on the detected levels of either of these organic acids (Fig 8). The largest (though not statistically significant) change in butyrate concentration was a decrease seen with the 1.0% bleach treatment. Given that this follows the same trend seen with diversity, it may indicate that this treatment is not fully adequate. The main contaminant from the pulses was identified as *Erwinia* and we would expect it to carry out a mixed acid fermentation common to members of the Enterobacteriaceae. Species and strains of *Erwinia* are known to vary in the relative amounts of succinate, formate, lactate, acetate, 2,3 butanediol and ethanol that they produce [44]. No significant amounts of succinate, formate or lactate were found in the fermentations, though all three are capable of being converted to other products by gut microbes [47, 48]. Alcohols were not measured. Given that acetate levels were not found to change this suggests that although some of the pulse contaminants could be detected in the colonic fermentations and had some impact on the community structure, it was insufficient to induce functional change at the level of acetate and butyrate production. Furthermore, the sterilization treatments themselves exhibited no significant detrimental impact on acetate and butyrate production.

### 3.4 Scanning electron microscopy of pulse flours

**Fig 9:**
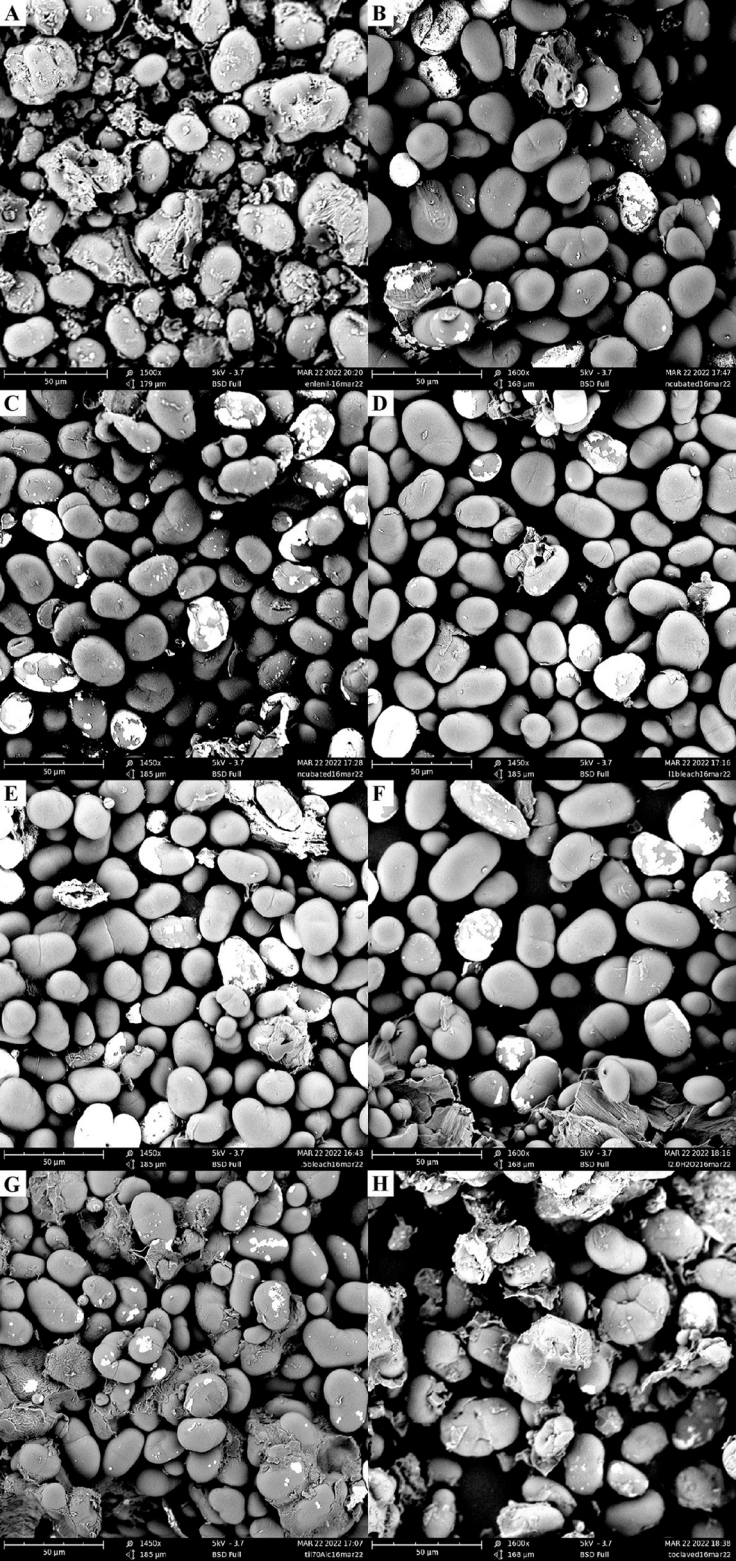
Scanning electron microscopy images of treated and untreated green lentil pulse flours at 50 μm scale. Treatments: (A) untreated control, not washed and not chemically treated (B) DI water washed 10 times and immediately dried, not incubated; (C) DI water incubated, then DI water washed 10 times and immediately dried; (D) Treated with bleach (1.0%) incubated, then DI water washed 10 times and immediately dried; (E) Treated with bleach (1.5%) incubated, then DI water washed 10 times and immediately dried; (F) Treated with hydrogen peroxide (2.0%) incubated, then DI water washed 10 times and immediately dried; (G) Treated with seventy percent alcohol (70%) incubated, then DI water washed 10 times and immediately dried; (H) autoclaved as a dry powder, then DI water washed 10 times and immediately dried.

As the major motivation for conducting these studies was to find ways to avoid damaging heat labile substances during in vitro investigation, assessment of the effects of the tested treatments on such substrates was needed. Scanning electron microscopy was used to evaluate the impact of the sterilization treatments on the pulse flours. All treatments showed similarly intact starch granules, with little damage or distortion detected (Fig 9, S1–S3 Figs). Only the pulse flour autoclaved as a dry powder showed minor signs of starch granule damage. Note that the standard autoclaving starch after incorporation into liquid media, destroys starch due to gelatinization. The gelatinization process is dependent on the presence of water and these pulse flours had a typical moisture content of about 8%. In the case of dry autoclaving, changes to the starch that take place are not necessarily destructive, but rather can increase its resistance to digestion [49]. Starch is the main fermentable carbohydrate present in these samples and there was not a significant increase in acetate or butyrate production in autoclaved samples, nor were there significant increases in known resistant starch degrading bacteria such as *Ruminococcus bromii* or *Bifidobacterium adolescentis* [8], but this possibility is a reason for caution when using this means of sterilization. Overall, it is possible that the minimal damage detected by the sterilization treatments could be due to the rigorous washing step each pulse underwent, as unwashed pulse (Fig 9A) did show physical components present that were absent in washed samples. Washing could potentially wash away damaged starch granules, giving the impression of insignificant damage from a treatment. However, additional processing of substrates, such as simulating upper intestinal tract digestion, is common before initiating in vitro colon fermentations, so it is likely that the removal of these components would happen anyway.

## 4. Conclusions

Here, plate counts, scanning electron microscopy, and in vitro simulated colonic fermentation (measuring microbial community composition and SCFA production) were used to examine the effects of non-thermal alternatives for reducing microbial loads in pulse flours for in vitro studies of food digestion by the human gut microbiota. While pulse microbes did not appear to influence organic acid levels during simulated colonic fermentation with unsterilized pulse flours, they were present at detectable levels suggesting they could exert some influence on these types of experiments. All treatments were effective at reducing microbial load, particularly the main contaminant *Erwinia*, though 70% alcohol did not reduce total counts of microbes below detectable levels. Only minor impacts were found on overall community structure by alpha and beta diversity though all sterilization treatments exhibited a minor shift in the community structure away from that of the unsterilized samples, probably due to the elimination of the pulse microbes. Only the dry autoclaving had any visually detectable effects on the pulse flours, while some concerns remain about the influence of this thermal treatment on starch structure. Thus, bleach and hydrogen peroxide treatments appear to be the most suitable overall. In terms of concentration, 2% hydrogen peroxide is highly effective in this context, with no detectable detrimental effects on the substrates. Likewise, 1.5% bleach was highly effective, with a greater reduction in *Erwinia* level compared to the 1.0% bleach and without the decrease in diversity or butyrate levels during simulated colonic fermentation seen for the 1.0%- concentration. Despite no detectable detrimental effect on substrates, oxidation of the substrates with these chemicals is possible, however, the extent of oxidation would be minimal with generally less than 2.0% of anhydrous glucose units undergoing substitution with a carbonyl or carboxyl group, while the main site of this reaction is in the amorphous region of starch [50, 51]. Thus, both bleach and hydrogen peroxide are appropriate choices to use for microbial load reduction in pulse flours and potentially other starchy food products prior to in vitro simulated colonic fermentation experiments.

## Supporting information

S1 Fig Scanning electron microscopy images of treated and untreated sprouted green lentil pulse flours at 50 μm scale. Treatments: (A) untreated control, not washed and not chemically treated (B) DI water washed 10 times and immediately dried, not incubated; (C) DI water incubated, then DI water washed 10 times and immediately dried; (D) Treated with bleach (1.0%) incubated, then DI water washed 10 times and immediately dried; (E) Treated with bleach (1.5%) incubated, then DI water washed 10 times and immediately dried; (F) Treated with hydrogen peroxide (2.0%) incubated, then DI water washed 10 times and immediately dried; (G) Treated with seventy percent alcohol (70%) incubated, then DI water washed 10 times and immediately dried; (H) autoclaved as a dry powder, then DI water washed 10 times and immediately dried.
(TIF)

S2 Fig Scanning electron microscopy images of chickpea pulse flour treatments at 50 μm scale. Treatments: (A) untreated control, not washed and not chemically treated (B) DI water washed 10 times and immediately dried, not incubated; (C) DI water incubated, then DI water washed 10 times and immediately dried; (D) Treated with bleach (1.0%) incubated, then DI water washed 10 times and immediately dried; (E) Treated with bleach (1.5%) incubated, then DI water washed 10 times and immediately dried; (F) Treated with hydrogen peroxide (2.0%) incubated, then DI water washed 10 times and immediately dried; (G) Treated with seventy percent alcohol (70%) incubated, then DI water washed 10 times and immediately dried; (H) autoclaved as a dry powder, then DI water washed 10 times and immediately dried.
(TIF)

S3 Fig Scanning electron microscopy images of field pea pulse flour treatments at 50 μm scale. Treatments: (A) untreated control, not washed and not chemically treated (B) DI water washed 10 times and immediately dried, not incubated; (C) DI water incubated, then DI water washed 10 times and immediately dried; (D) Treated with bleach (1.0%) incubated, then DI water washed 10 times and immediately dried; (E) Treated with bleach (1.5%) incubated, then DI water washed 10 times and immediately dried; (F) Treated with hydrogen peroxide (2.0%) incubated, then DI water washed 10 times and immediately dried; (G) Treated with seventy percent alcohol (70%) incubated, then DI water washed 10 times and immediately dried; (H) autoclaved as a dry powder, then DI water washed 10 times and immediately dried.
(TIF)
