# Supplementary figures and images for: Evaluating the efficacy of non-thermal microbial load reduction treatments of heat labile food components for in vitro fermentation experiments
Source: PLoS One. 2023 Mar 21;18(3):e0283287. doi: 10.1371/journal.pone.0283287 (PMC10030034; doi:10.1371/journal.pone.0283287)

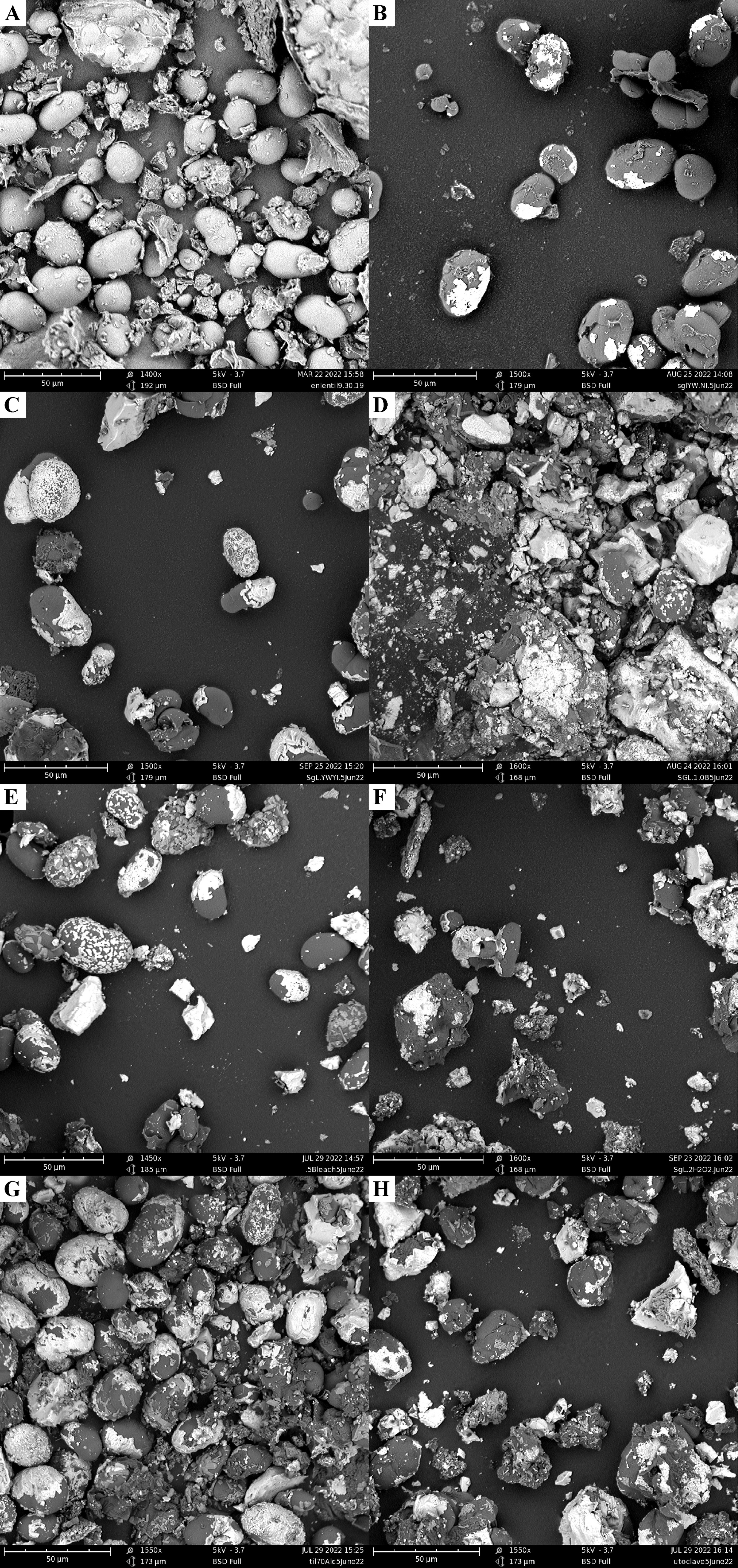

Supplement: S1 Fig — Treatments: (A) untreated control, not washed and not chemically treated (B) DI water washed 10 times and immediately dried, not incubated; (C) DI water incubated, then DI water washed 10 times and immediately dried; (D) Treated with bleach (1.0%) incubated, then DI water washed 10 times and immediately dried; (E) Treated with bleach (1.5%) incubated, then DI water washed 10 times and immediately dried; (F) Treated with hydrogen peroxide (2.0%) incubated, then DI water washed 10 times and immediately dried; (G) Treated with seventy percent alcohol (70%) incubated, then DI water washed 10 times and immediately dried; (H) autoclaved as a dry powder, then DI water washed 10 times and immediately dried. (TIF) [file pone.0283287.s001.tif]

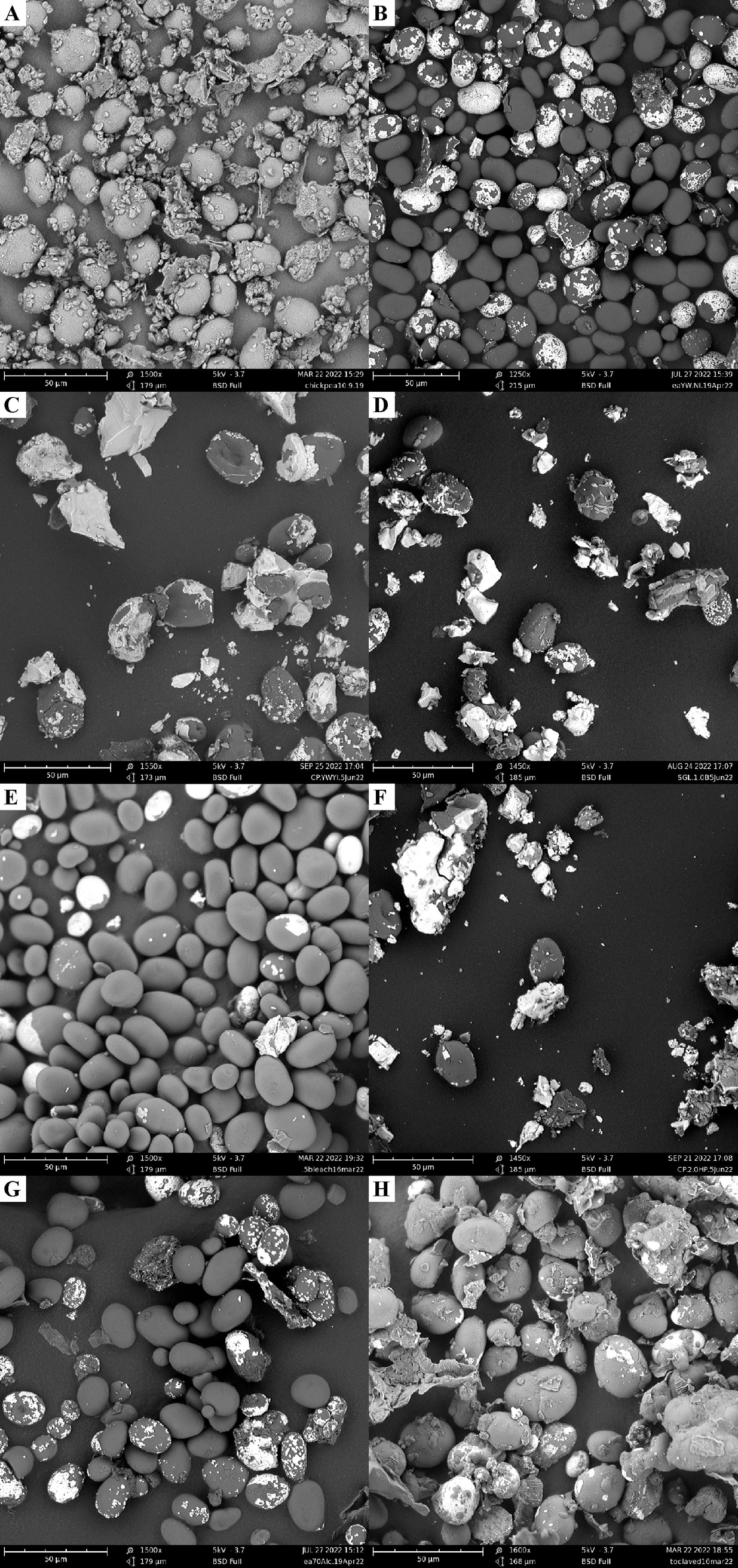

Supplement: S2 Fig — Treatments: (A) untreated control, not washed and not chemically treated (B) DI water washed 10 times and immediately dried, not incubated; (C) DI water incubated, then DI water washed 10 times and immediately dried; (D) Treated with bleach (1.0%) incubated, then DI water washed 10 times and immediately dried; (E) Treated with bleach (1.5%) incubated, then DI water washed 10 times and immediately dried; (F) Treated with hydrogen peroxide (2.0%) incubated, then DI water washed 10 times and immediately dried; (G) Treated with seventy percent alcohol (70%) incubated, then DI water washed 10 times and immediately dried; (H) autoclaved as a dry powder, then DI water washed 10 times and immediately dried. (TIF) [file pone.0283287.s002.tif]

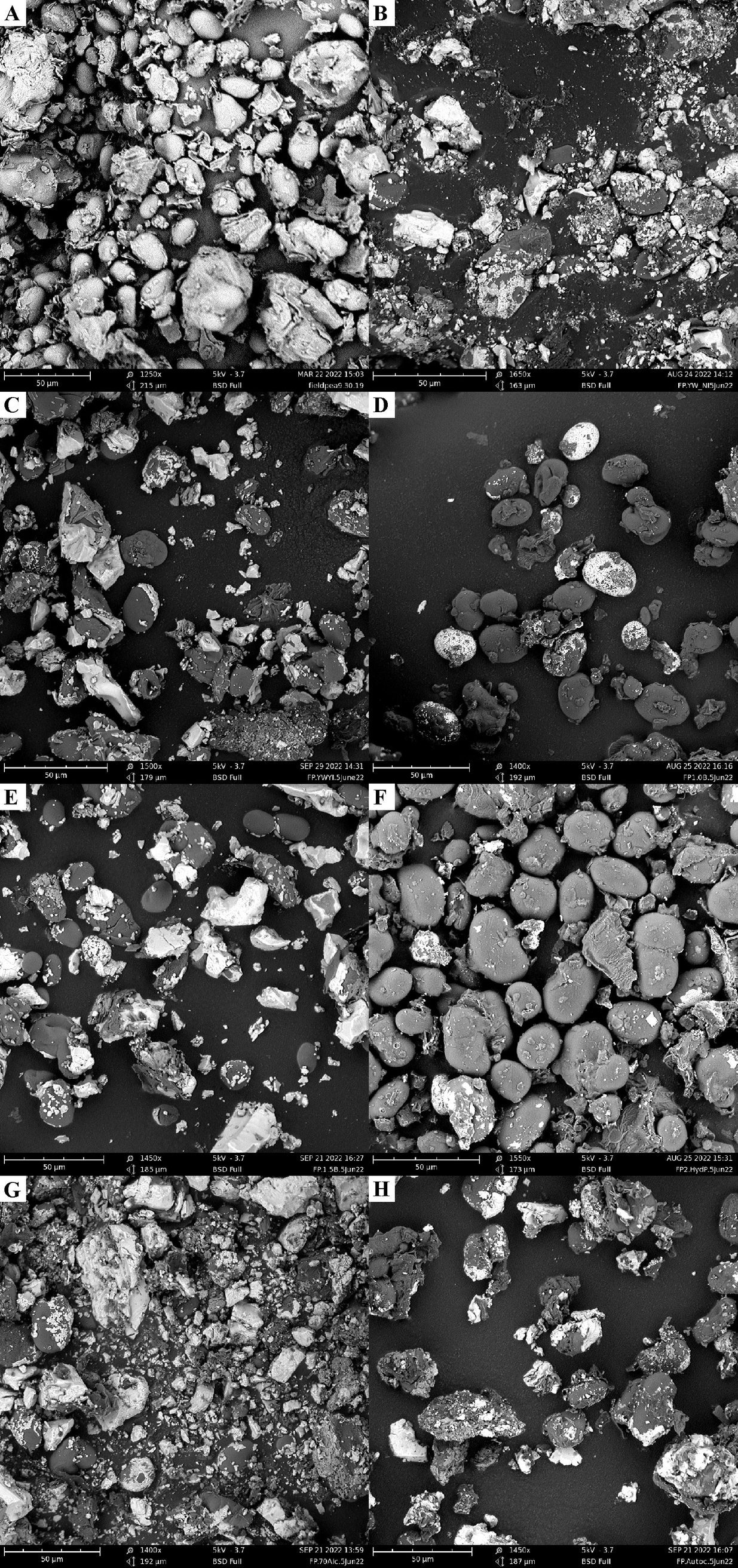

Supplement: S3 Fig — Treatments: (A) untreated control, not washed and not chemically treated (B) DI water washed 10 times and immediately dried, not incubated; (C) DI water incubated, then DI water washed 10 times and immediately dried; (D) Treated with bleach (1.0%) incubated, then DI water washed 10 times and immediately dried; (E) Treated with bleach (1.5%) incubated, then DI water washed 10 times and immediately dried; (F) Treated with hydrogen peroxide (2.0%) incubated, then DI water washed 10 times and immediately dried; (G) Treated with seventy percent alcohol (70%) incubated, then DI water washed 10 times and immediately dried; (H) autoclaved as a dry powder, then DI water washed 10 times and immediately dried. (TIF) [file pone.0283287.s003.tif]
